# The current status of emergency departments in secondary emergency medical institutions in Japan: a questionnaire survey

**DOI:** 10.1186/s12245-023-00513-0

**Published:** 2023-06-23

**Authors:** Toshiki Sera, Norio Otani, Hideo Bannai, Takanori Hasegawa, Takehiro Umemura, Hideki Honda, Akio Kimura

**Affiliations:** 1 Department of Emergency and Critical Care Medicine, Graduate School of Biomedical and Health Sciences, Hiroshima University, 1-2-3 Kasumi, Minami-Ku, Hiroshima, 734-8551 Japan; 2 Department of Emergency and Critical Care Medicine, St. Luke’s International Hospital, 9-1 Akashi-Cho, Chuo-Ku, Tokyo, 104-8560 Japan; 3 Division of Data Science Algorithm Design and Analysis M&D Data Science Center, Tokyo Medical and Dental University, 2-3-10 Kandasurugadai, Chiyoda-Ku, Tokyo, 101-0062 Japan; 4 Department of Integrated Analytics M&D Data Science Center, Tokyo Medical and Dental University, 2-3-10 Kandasurugadai, Chiyoda-Ku, Tokyo, 101-0062 Japan; 5 Department of Emergency and Critical Care Medicine, Graduate School of Medicine, University of the Ryukyus, 207 Uehara, Nishihara-Cho, Okina, 903-0215 Japan; 6 Department of Emergency and Critical Care Medicine, Yokosuka General Hospital Uwamachi, 2-36 Uwamachi, Yokosuka-City, Kanagawa 238-0018 Japan; 7 Department of Emergency Medicine and Critical Care, Center Hospital of the National Center for Global Health and Medicine, 1-21-1 Toyama, Shinjuku-Ku, Tokyo, 162-8655 Japan

**Keywords:** Secondary emergency medical institution, Emergency medical system, Emergency department, Japan, Emergency care

## Abstract

**Background:**

While emergency medicine (ER)-based emergency care is prevalent in many countries, in Japan, the “department-specific emergency care model” and the “emergency center model” are mainstream. We hypothesized that many secondary emergency medical institutions in Japan have inadequate systems. Using a questionnaire, we investigated the status of and problems in the emergency medical services system in secondary emergency medical institutions in Japan. Until date, there has not been an exhaustive survey of emergency facilities on a countrywide scale. The main objective of this study was to investigate problems in the Japanese emergency medical services system and thereby improve optimal care for emergency patients.

**Results:**

A nationwide questionnaire survey involving 4063 facilities (all government-approved emergency medical facilities certified by prefectural governors) in Japan was conducted. Of the facilities that responded, all secondary emergency facilities were included in the analysis. Responses from 1289 facilities without a tertiary emergency medical care center were analyzed. Among them, 61% (792/1289) had ≤ 199 beds, and 8% were emergency department specialty training program core facilities. Moreover, 42% had an annual patient acceptance number of ≤ 500, 19% did not calculate the number of acceptances, 29% had an acceptance rate of ≥ 81%, and 25% had an acceptance rate of 61–80%. Pregnant women (63%) and children (56%) were the major types of patients that affected the acceptance rate. Factors affecting facilities with a response rate of 81% or higher were “hospitals designated for residency training” and “facilities making some efforts to improve the quality of emergency care and the emergency medical system” (logistic analysis, *P* < .001).

**Conclusion:**

Relevant authorities and core regional facilities should consider and implement specific measures for regions and hospitals with a shortage of emergency medicine specialists and physicians (e.g., development of ER-based emergency medicine and provision of education). This study may lead to further improvement in the optimal care of emergency patients through the nationwide establishment of the proposed measures as well as through grouping and integrating the structures and systems in emergency and other medical facilities.

## Background

There are several issues around the emergency systems in Japan, such as multi-specialty staffing, insufficient numbers of emergency physicians, and transfer of older patients in nursing homes during emergencies [1]. The increase in emergency patients in an aging society and increase in ambulance dispatch and diversion make hospital selection more difficult [1].

Emergency hospitals in Japan are classified in three levels according to the severity of patients. Primary emergency medical institutions treat outpatients who do not require hospitalization, secondary emergency medical institutions treat patients who require hospitalization, and tertiary emergency medical institutions treat patients who are so serious that they need to be managed in an intensive care unit [2]. The most severely ill patients are taken by ambulance to a tertiary care hospital, while other moderately ill patients are taken by ambulance to a secondary care hospital. The secondary emergency medical institutes are responsible for most emergency transports. The emergency medical services (EMS) team contacts the hospital, which will decide whether to accept and transport the patient. They are licensed by the prefecture but are not legally bound to accept patients; hence, how things “should work” is a social issue.

In Japan, the “emergency center-type emergency medical care model” (emergency physicians specially trained to deal with critically ill patients, where tertiary medical institutes) and the “department-specific emergency medical care model,” unique to the country, have been the main models in operation for a long time [3]. The “ER-based emergency medicine model,” newly added to Japan’s emergency medical care delivery system, is the third model [3]. Since 2004, ER-based emergency medicine has been the subject of active discussion at academic conferences in Japanese Association for Acute Medicine (JAAM). Contrastingly, in the “department-specific emergency medical care model,” physicians are generally non-emergency medicine specific trained; therefore, education for standardization of treatment and quality improvement within the hospital and in the community are desired.

No detailed survey has been conducted regarding the status of secondary emergency medical institutions in Japan. Thus, there is a lack of visibility of issues that need to be resolved by the academic community, government (include local governments), and local hospitals certified emergency hospital in the region. We hypothesized that secondary emergency medical institutions, which are responsible for most emergency transportation in Japan, accept many ambulances despite their systems being inadequate. Therefore, we surveyed secondary emergency medical institutions and clarified the status and problems of the emergency care system.

## Results

**Fig. 1:**
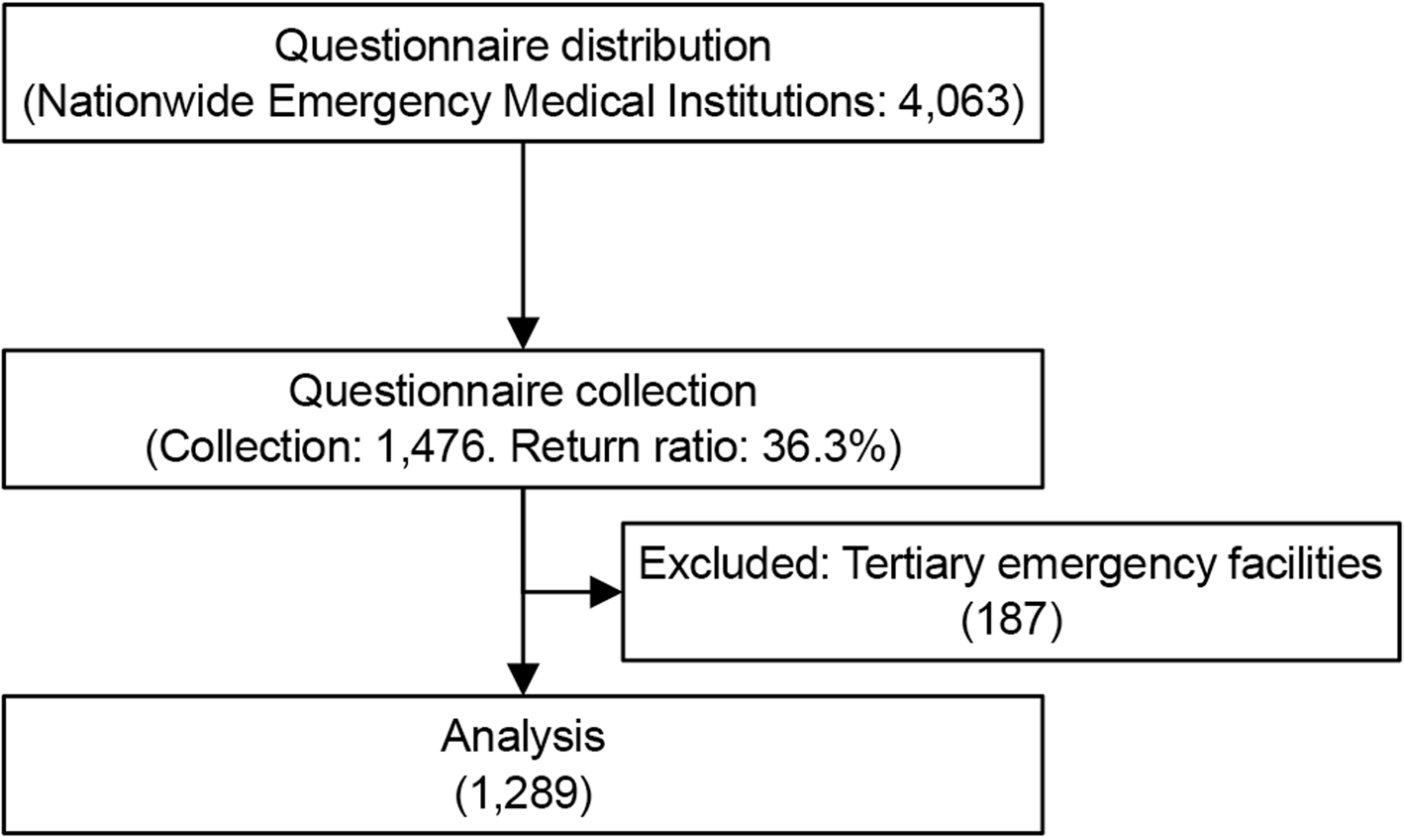
Survey and analysis flow

A total of 1476 facilities responded (response rate: 36.3%), and 1289 facilities were included in the analysis after excluding facilities with tertiary emergency centers (Fig. 1).

### Facility characteristics

**Fig. 2:**
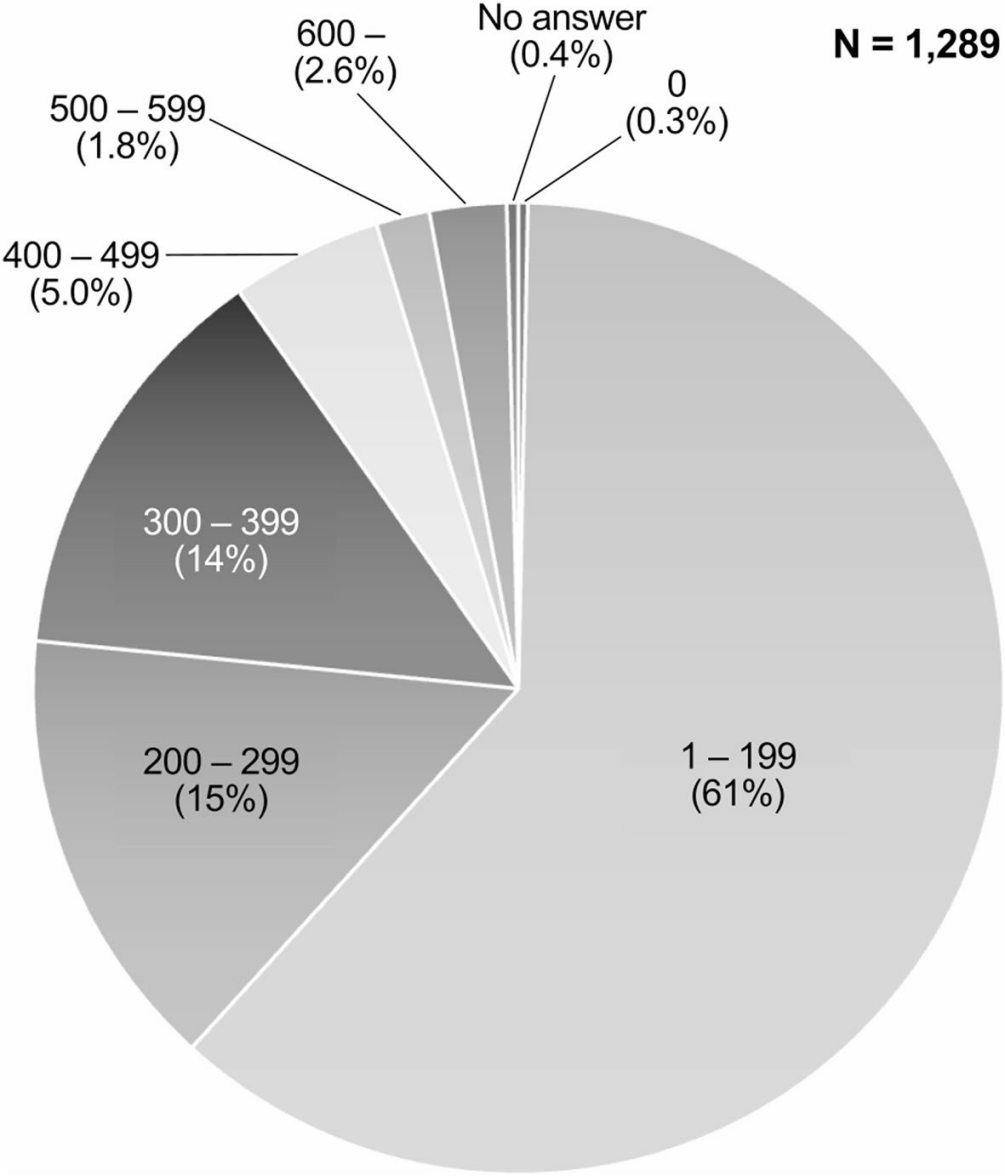
Number of beds at responding facilities

Of the responding facilities, hospitals with 199 beds or less accounted for 61% (792/1289), making up the largest group (Fig. 2). In total, 23% (293/1289 facilities) were core clinical training hospitals designated by the Ministry of Health (where residents are supervised), Labour and Welfare for initial clinical training, 32% (413/1289 facilities) were cooperative clinical training hospitals (hospitals that are part of the training program of core clinical training hospitals), and 41% (529/1289 facilities) had no such designations. The number of core facilities for emergency medicine specialty training programs was 1.9% (25/1289).

### Characteristics of the emergency department

**Fig. 3:**
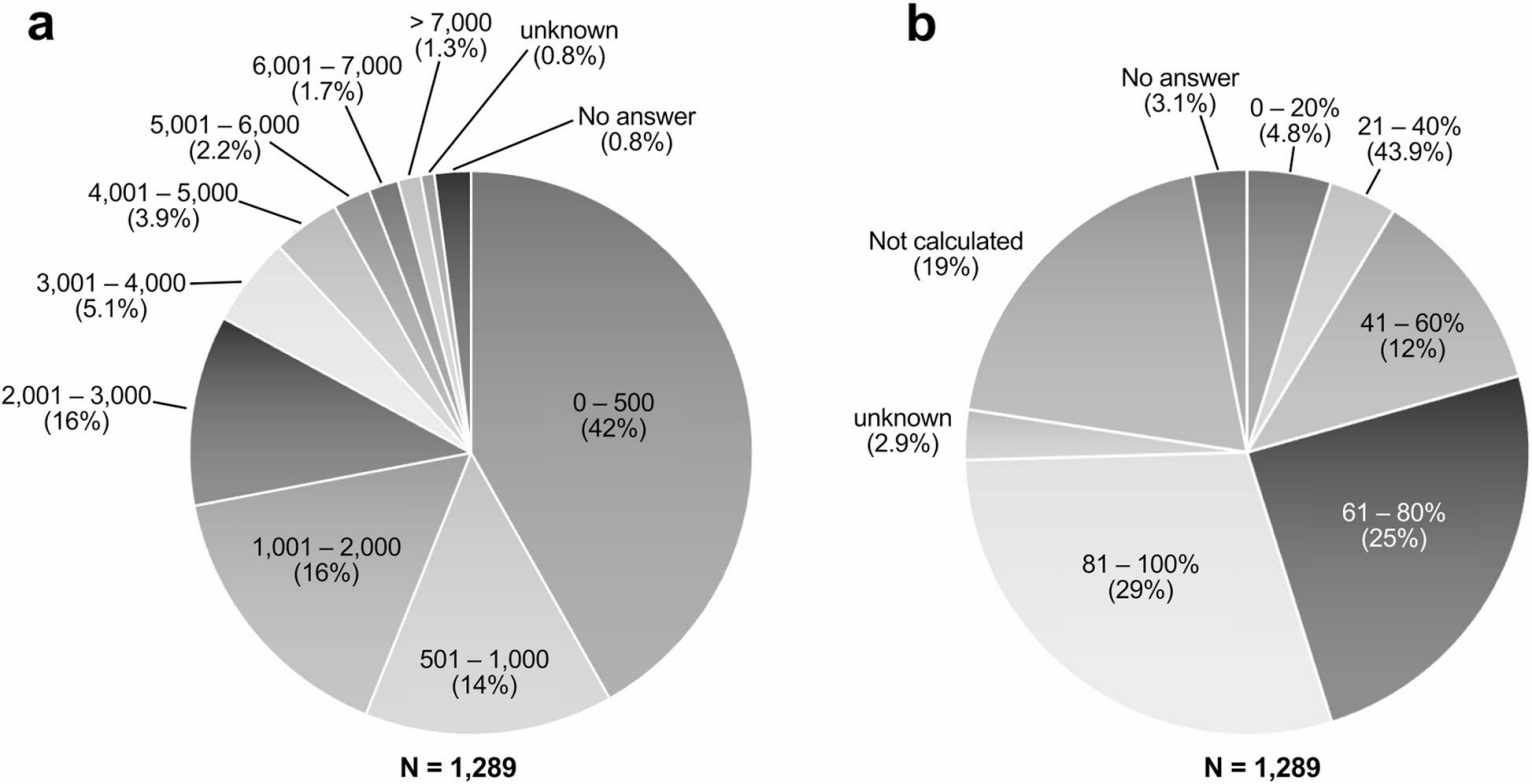
Characteristics of the emergency departments at the responding facilities. **a** Number of injured and ill patients received by emergency medical services during the year (only those accepted through communication from the fire department, such as firefighting ambulances and firefighting disaster prevention helicopters). **b** Demand response rate (secondary emergency only)

Altogether, 68% (882/1289 facilities) responded to patients 24 h a day, 365 days a year; 17% (222/1289 facilities) responded on a rotating basis, and 3.6% (46/1289 facilities) responded only during weekdays. The annual number of emergency casualties was 0–500 in 42% (539/1289 facilities) and 501–1000 in 14% (184/1289 facilities; Fig. 3a). The largest number of facilities (29%; 370/1289 facilities) had an annual acceptance rate (accepting patients from pre-hospital providers in ambulances) of 81–100% (Fig. 3b). The number of facilities, which were “clinical training hospitals;” “making efforts to improve the quality of emergency care and the local emergency medical care system;” had “more than 200 beds,” “physicians available at all times;” “pharmacists, clinical technologists, and radiologists available at all times;” had made “efforts to improve the quality of emergency care and the local emergency medical system,” and had “core facilities of emergency department training programs” were significantly higher (data not shown).

### System for accepting patients for emergency transportation

Of the responding facilities, 18% (225/1289 facilities) included non-physicians who accepted a request for transportation from the fire department; 69% (885/1289 facilities) recorded the reason for not accepting the request, 24% (304/1289 facilities) did not, 4.1% (53/1289 facilities) recorded the reason as “other,” and 3.7% (48/1289 facilities) recorded “no answer.” Among hospitals with clinical training, the number of institutions that recorded the reason was significantly higher (data not shown).

The presence or absence of rules in selecting a hospital in the area was “Yes” for 24% (304/1289 facilities), “No” for 45% (580/1289 facilities), “Don’t know” for 28% (366/1289 facilities), and 3.0% (39/1289 facilities) did not respond. The factors that influenced (or made facilities hesitate to accept) were as follows: pregnant women (63%, 807/1289), children (56%, 719/1289), drug addicts (50%, 643/1289), patients with a history of mental disorder (50%, 639/1289), alcoholics (36%, 462/1289), trauma patients (21%, 266/1289), patients with fever (19%, 239/1289), others (12%, 149/1289), patients living alone (3.0%, 39/1289), and patients dependent on welfare payments (1.3%, 17/1289); 3.1% did not respond.

### Personal aspects in the emergency department

**Fig. 4:**
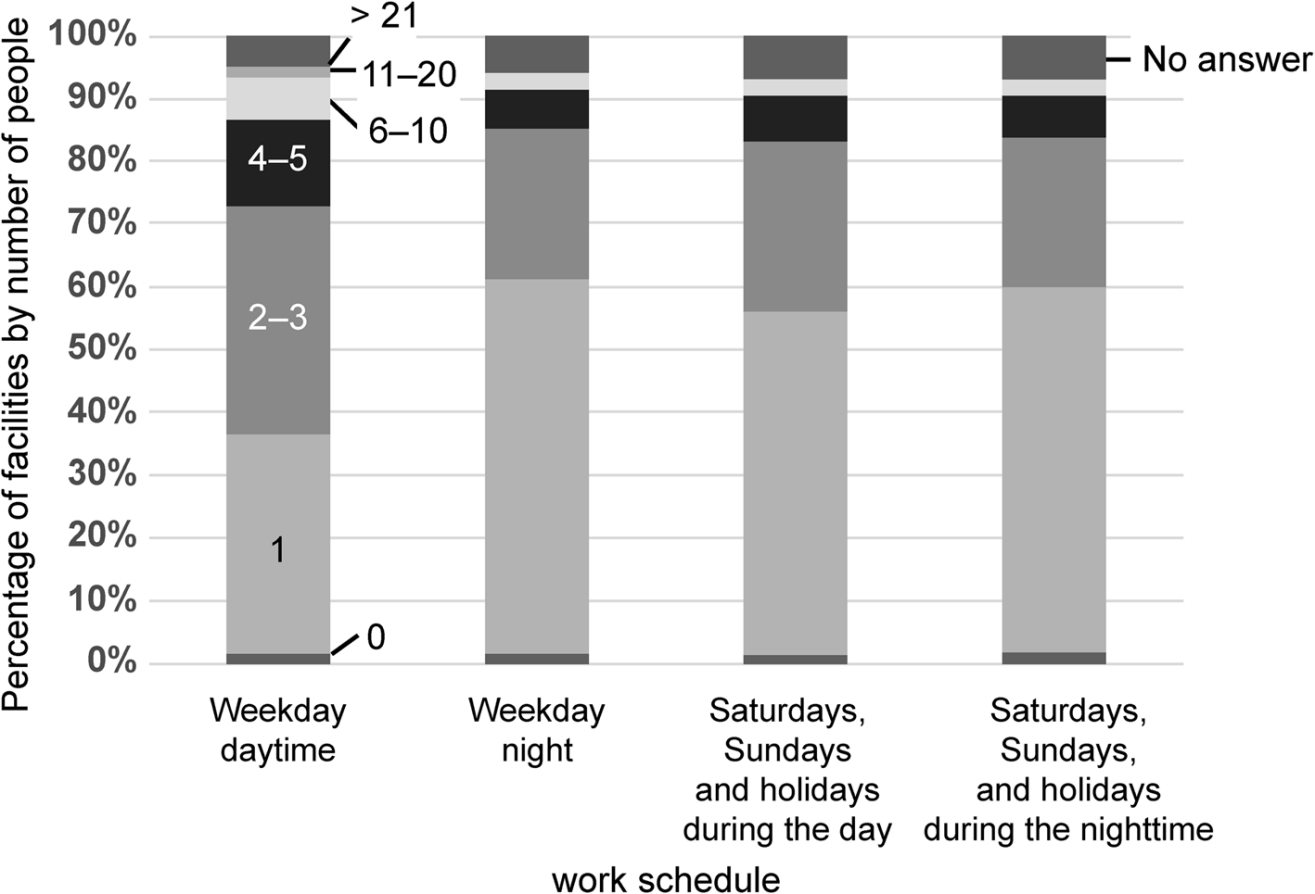
Number of physicians treating patients transported by ambulance. (Refers to pre-determined shifts and ambulance duties. May be crossed with wards. Residents are included. In the case of rotation, the current day is assumed to be the day of the rotation)

**Fig. 5:**
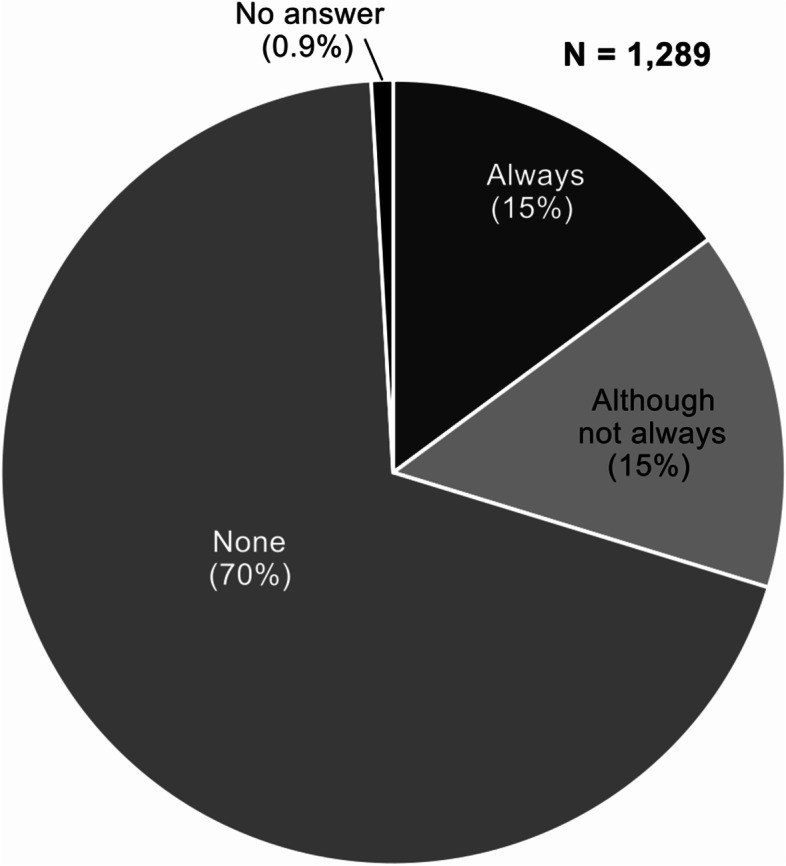
Availability of dedicated emergency department physicians (The term “full-time” here is defined as those who devote 80% or more of their working hours to emergency department care in one shift, regardless of whether they work full-time or part-time.)

Except during weekdays, the number of physicians in charge of treating patients transported by ambulance ranged from 0 to 1 in more than half of the facilities (Fig. 4). The number of dedicated emergency department physicians was the highest (70%, 897/1289 facilities), followed by “no dedicated emergency department physicians” (Fig. 5).

Facilities that “always had a dedicated emergency physician available” were significantly more likely to be “clinical training hospitals” than non-clinical training hospitals (Fisher’s exact test, *P* < 0.001). The number of dedicated physicians (no full-time or part-time, month listed, no duplicates) at the 381 facilities that reported having a dedicated physician was 1–5, 72% (266/367); 2–3, 15% (54/367); 4–5, 4.4% (16/367); 6–10, 3.5% (13/367); and 11–20, 4.9% (18/367). The number of emergency department specialists among the dedicated physicians was 0, 27% (99/361); 1, 33% (119/361); 2–3, 25% (89/361); 4–5, 8.3% (30/361); 6–10, 5.5% (20/361); and 11–20, 1.1% (4/361). Among the facilities with dedicated physicians, 62% (224/360) had 0 female physicians, 23% (83/360) had 1, 10% (36/360) had 2–3, 2.2% (8/360) had 4–5, 1.9% (7/360) had 6–10, and 0.6% (2/360) had 11–20. The number of dedicated physicians and emergency medicine physician specialists was significantly higher in the clinical training hospitals (*P* = 0.009 and *P* < 0.001, respectively), while there was no significant difference in the number of female physicians (Fisher’s exact test, *P* = 0.143).

In the system for receiving ambulances, 41% (527/1274) of clinical technologists were always involved, 44% (558/1274) were conditionally involved, and 15% (189/1274) were not involved. Among pharmacists, 25% (314/1274) were always involved, 45% (574/1274) were conditionally involved (depending on the time of day, and so on), and 30% (386/1274) were not involved. Among radiologists, 56% (710/1278) were always involved, 37% (475/1278) were conditionally involved (depending on the time of day), and 7.2% (93/1278) were not involved. Involvement was significantly higher in training hospitals for all occupations (Fisher’s exact test, *P* < 0.001).

### Quality of medical care and educational system in the emergency department

Of all facilities, 65% (830/1279 facilities) were taking initiatives to improve the quality of emergency care and the local emergency medical system, 35% (453/1279) were not, and 1% (10/1289) did not respond to this parameter. Facilities with initiatives were more likely to be clinical training hospitals (78% (558/715) vs. 46% (238/523); Fisher’s exact test, *P* < 0.001). Of the facilities with initiatives, 64% (529/830) were involved in internal validation (departmental review, M&M, and so on), 55% (453/830) participated in medical control councils, and 20% (169/830) held case-review meetings among local hospitals. The number of facilities not involved in any initiative was 35% (449/1279 facilities). Of the facilities that were not involved in such efforts, 37% (164/446) cited “no time,” 34% (15/446) “don’t know what to do,” 15% (68/446) “don’t need to,” and 21% (92/446) “other” as reasons.

Regarding whether physicians (excluding residents) who respond to emergency patients take measures based on standardized initial care tools (Japan Advanced Trauma Evaluation and Care [JATEC], Immediate Cardiac Life Support [ICLS], resuscitation training courses offered by the JAAM), and so on), 43% (558/1289) answered “yes,” 17% (220/1289) answered “no,” 17% (214/1289) answered “neither,” 22% (281/1289) answered “don’t know,” and 1.2% (16/1289) did not respond. More facilities acted based on the initial care standardization tool in clinical training hospitals (83% (398/478) vs. 50% (135/269), Fisher’s exact test, *P* < 0.01).

Basic life support (BLS) was recommended to physicians and nurses involved in emergency care by 58% (751/1289) of the facilities, ICLS by 40% (513/1289), JATEC by 16% (202/1289), and Japan Prehospital Trauma Evaluation and Care by 15% (193/1289), and “Not recommended” 33% (427/1289), respectively. The number of annual training sessions (ICLS, BLS, and so on) related to first aid and resuscitation (in 2019) conducted were 10 or more times in 8.2% (106/1289) of the facilities, 5–10 times in 9.4% (122/1289), 1–5 times in 60% (779/1289), and none in 21% (270/1289).

### Characteristics of facilities with an annual demand rate of 81–100

Multivariate logistic regression analysis was conducted using the following explanatory variables: whether the facility has an annual demand rate of 81–100% as the objective variable, and factors that could affect demand, such as “core emergency department training program,” “facilities with 200 or more beds,” “clinical training hospitals,” “facilities making efforts to improve the quality of emergency care at the facility and the emergency medical system in the community,” and “facilities that always have a dedicated physician available.” A multivariate logistic regression analysis was conducted using “facilities that always have a dedicated physician available” as an explanatory variable. “Clinical training hospitals” and “facilities that are making efforts to improve them” were identified as factors that significantly influenced the objective variable (Table 1).

**Table 1:** Factors affecting annual response rate of 81% or more facilities (logistic analysis)

	Coefficient	odds ratio	95% confidence interval			*P*
Core facility for emergency medicine specialists	0.833	2.3	0.808	ー	6.543	.119
Facilities with more than 200 beds	0.335	1.398	0.996	ー	1.964	.053
Designated clinical training hospitals	0.609	1.838	1.306	ー	2.589	< .001
Working to improve the quality of emergency care and the emergency medical system	0.951	2.588	1.799	ー	3.723	< .001
There is always a dedicated doctor in the emergency room	0.379	1.461	0.99	ー	2.157	.056

### Requests for each organization (free-text)

#### Requests for JAAM

A total of 299 facilities (23%) provided responses, and 120 facilities responded with “none in particular.” The remaining 179 facilities that responded with specific descriptions were characterized as “clinical training hospitals” (*P* < 0.001), “core facility of the emergency training program” (*P* = 0.016), “rotation participating hospitals” (*P* = 0.017), facilities with “more than 200 beds” (*P* < 0.001), “annual demand rate of 81% or more” (*P* = 0.004), and “making some efforts to improve the quality” (*P* < 0.001). A total of 198 opinions from 179 facilities were categorized (Table 2).

**Table 2:** Requests (free description) from secondary emergency medical institutions—requests to the Japanese Association for Acute Medicine (3 or more comments in bold)

Number of opinions	
**29**	**Provide education to secondary medical institutions**
**26**	**Increase in the number of emergency physicians, training of specialist physicians, sufficiency of emergency physicians at core hospitals, and elimination of unequal distribution of emergency physicians**
**26**	**Lobbying the national government**
**14**	**Obtaining or renewing an emergency specialist medical license**
**12**	**Educating the public**
**10**	**Attention and support for secondary emergency medical facilities**
**9**	**Leading network building and mutual hospital cooperation**
**6**	**Evaluation**
**6**	**Contents related to medical specialists**
**5**	**Workplace reforms**
**4**	**Training and competence of emergency physicians**
**3**	**Eliminate physician shortage and physician maldistribution**
**3**	**Improvement of the status of emergency physicians**
**3**	**Training and education of emergency nurses (including certified)**
**3**	**Development of psychiatric emergency services**
**3**	**Emergency system (primary to tertiary)**
**3**	**Developing guidelines and guidelines as an academic society independently in Japan**
**3**	**Utilization of emergency medical technicians**
**3**	**Contents related to emergency medicine**
**3**	**Content related to the survey**
2	Active support to facilities with emergency physicians
2	Continue to hold scientific meetings on the web owing to my busy schedule and being alone
2	Promote research on firefighting and medical control
2	Consideration of charging for ambulance service
2	About the Society’s Certified Resuscitation Training Course
13	Others

The most common request was “providing education to secondary emergency medical institutions.” Not all such respondents were “core facilities of the emergency department specialty training program;” 54% (16/29) were “clinical training hospitals,” 38% (11/29) had “more than 200 beds,” and 59% (17/29) were “making some efforts to improve the quality.” Such a response was more common in facilities with higher annual response rates.

Subsequently, the most common requests included an increase in the number of emergency physicians, training of specialists, and filling and eliminating the maldistribution of emergency physicians at core hospitals. All the facilities that submitted this request were also not “core facilities of the emergency department specialty training program,” with 77% (20/26) of them being “clinical training hospitals,” 73% (19/26) having “more than 200 beds,” and 96% (25/26) “making some efforts to improve the quality of emergency care and the local emergency medical system.” Such a response was more common in facilities with higher annual response rates.

#### Requests for nearby secondary medical institutions

Altogether, 420 facilities (33%) provided responses, and 105 cited “none.” The remaining 315 facilities that responded with specific descriptions were characterized as “clinical training hospitals,” “core facilities of emergency training programs,” “rotation participating hospitals,” facilities with “more than 200 beds,” “an annual demand rate of 81% or more,” and “making some efforts to improve the quality” (*P* < 0.05). Each statement was categorized by item (328 opinions from 315 facilities, including 13 duplicate opinions within one facility; Table 3).

**Table 3:** Requests (free description) from secondary emergency medical institutions**—**requests to nearby secondary medical institutions (3 or more opinions in bold)

Number of opinions	
**147**	**Prompt patient acceptance and improved level of care**
**107**	**Sharing of roles among hospitals and medical regions, enhancement of cooperation, and establishment of a cooperative system through information sharing**
**44**	**Works well**
**9**	**Yes (unspecified)**
**7**	**Increase in the number of secondary medical institutions**
**4**	**No other secondary hospitals**
**4**	**Increase in the number of physicians and nurses**
**3**	**Maintain and increase the number of hospitals on rotation**
1	Hospital consolidation and reorganization
1	Our hospital is located on the border between prefectures; however, it is difficult for ambulances to cross the border between prefectures
1	I would like a report on the outcome of referred patients

The most common request was “improvement of the system and level of care for the prompt acceptance of patients.” Of the facilities that made this request, 69% (101/147) were “clinical training hospitals,” 7% (10/147) “core facilities of emergency department specialty training programs,” 56% (82/147) “hospitals with 200 or more beds,” and 75% (110/147) “making some efforts to improve the quality.” The higher the annual demand rate, the more often this request was made.

Subsequently, the most common request was “sharing roles, enhancing cooperation among hospitals and medical regions, and building a cooperative system through information sharing.” All but one of the facilities that submitted this request were not “core facilities of the emergency department training program; 74% (79/107) were “clinical training hospitals,” 53% (57/107) were “more than 200 beds,” and 80% (86/107) were “making some efforts to improve the quality,” with higher annual response rate indicating more responses in this category.

#### Requests for nearby tertiary medical institutions

Among the 408 facilities (32%) 113 cited “none.” The remaining 295 facilities that responded with specific descriptions were characterized as “clinical training hospitals,” facilities with “more than 200 beds,” “annual response rate of 81% or more,” and “making some efforts to improve the quality” (*P* < 0.001). There was no significant difference between the “core facilities of emergency training programs” and “rotating participating hospitals.” Each statement was categorized by item (310 statements from 295 facilities, including 15 duplicate statements within one facility; Table 4).

**Table 4:** Requests (free description) from secondary emergency medical institutions**—**requests to nearby tertiary medical institutions (3 or more opinions in bold)

Number of opinions	
**141**	**Patient acceptance requests** Smooth acceptance of patients who have been transferred from one hospital to another, who are seriously ill or difficult to accept, or who have been seen in the hospital before
**70**	**It’s working, thanks**
**28**	**Strengthening of cooperation between secondary and tertiary medical institutions** (a system that is easy to contact, easy to consult, easy to request, sharing of information such as medical records and images, and visualization throughout the community)
**13**	**No tertiary care facilities nearby** (2 of them are too far away to transport)
**13**	**Yes (unspecified)**
**10**	**Strengthening of systems within tertiary care institutions** (4 of them: securing vacant beds, expansion of beds), **strengthening of cooperation among tertiary care institutions**
**6**	**Don’t want them to take all my patients**
**4**	**Want overtriage to be allowed**
**4**	**Provision of guidance and education by tertiary care institutions** (Guidance through image viewing, collaboration with secondary medical institutions to foster emergency care, and holding and disseminating training)
**4**	**Dispatch of physicians to secondary medical institutions**
**3**	**Use downstream transport to secondary institutions to free-up beds for lifesaving and make room for tertiary cases**
**3**	**Secondary and tertiary hierarchy unclear (perhaps a future issue)**
2	Establish a regional emergency medical care system centered on tertiary medical institutions (Leadership of secondary medical institutions, designation/allowance of assistant tertiary medical institutions)
2	Education of attitudes and dispositions of staff of tertiary care institutions
1	Nighttime otolaryngology, ophthalmology, orthopedics, and dermatology to be rotated
1	Concentrating mostly on grants and other support for COVID19
1	collaborate with other departments in the hospital
1	Survival of the medical institution
1	Aggregation of emergency physicians and emergency patients
1	Reporting outcomes of referred patients
1	Come and pick patients up

The most common request was to improve the patient’s acceptance. Of the facilities that made this request, 68% (96/141) were “clinical training hospitals,” 2.9% (41/141) were “core facilities of emergency specialty training programs,” 46% (65/141) mentioned having “more than 200 beds,” and 80% (113/141) were “facilities that are making some efforts to improve the quality.” Facilities with higher annual demand rates indicated more responses in this category.

## Discussion

Regarding the annual number of emergency medical transports, “secondary emergency medical institutions are responsible for most emergency medical services” [4]. However, there has been no previous investigation of the status of such a large number of emergency medical facilities as in this study. This study partially revealed the status of emergency care systems at designated emergency medical institutions in Japan.

The results support our hypothesis that secondary emergency medical institutions accept many ambulances despite their systems being inadequate, in terms of a lack of full-time emergency physicians. Thus, they could be making requests to academic societies and others.

The survey revealed the current human and qualitative plight in secondary emergency medical institutions. Regarding human resources, while the number of emergency physicians is insufficient even at facilities that provide ER-type emergency care [5], the survey revealed that only a few hospitals of notified emergency medical institutions nationwide have relevant emergency physicians on duty, and many emergency transport patients are still treated by physicians from different departments and by residents. Many requests were made in the free-response column regarding a lack of human resources. Additionally, the survey revealed that the quality of emergency care was not being adequately maintained, considering the status of standardization of initial care and the fact that 34% (152 facilities) of respondents were “unsure what to do” when asked about “efforts to improve the quality of emergency care and the local emergency medical care system.”

The current results revealed the human resource and quality limitations of many notified emergency medical institutions, as they do not have dedicated emergency physicians. The limitations of the “individual department type emergency medicine model.” In studies in other countries, a lack of emergency medical residencies was also noted for bedside teaching [6]. It could be a characteristic of Japan given that many in this study called for inter-hospital collaboration. Many medical institutions have requested prompt patient acceptance and improved level of care, sharing of roles among hospitals and medical regions, enhancement of cooperation, and establishment of a cooperative system through information sharing to secondary medical institutions. This also includes acceptance of patients who have been transferred from one hospital to another, who are seriously ill or difficult to accept, or who have been seen in the hospital previously to tertiary institutions. More interestingly, JAAM is being asked not only to provide education to secondary EMS providers but also to give attention, support, and leadership in networking and mutual hospital cooperation to secondary medical institutions.

Regarding the issues surrounding emergency medical care, support measures are required to raise the level of response capability of secondary emergency medical institutions and enable them to accurately fulfill their roles [1, 3]. In addition, some have noted that policy consideration should be given to the current situation in which physicians with non-emergency specialties are broadly responsible for emergency services [7]. Given the increased demand for emergency care and the estimated supply and demand for physicians with relevant specialties, “emergency care will be difficult unless all physicians are available,” and “a cooperative relationship between physicians with emergency specialties and those with non-emergency specialties will be important in the future of emergency care” [8].

Further, emergency medicine residents must rotate not only between tertiary facilities but also between primary and secondary facilities to gain comprehensive experience. Some have noted the necessity of “a system that puts to practice a multi-specialty staffing model in designated tertiary emergency care centers, or the development of a single-specialty staffing model in the designated primary and secondary emergency care centers or possibly even in tertiary emergency care centers. This systemic change will subsequently reflect the needs of Japan’s super-aging society” [1].

The human plight and the shortcomings of many emergency medical institutions without dedicated emergency department physicians or emergency physicians in the survey results suggest the limitations of the “individual department type emergency medical care model.” In addition, many respondents expressed their expectations for “ER-based emergency medicine” in their free responses to the survey. Therefore, it is desirable to further increase the number of the number of emergency physicians, facilities that provide “ER-type emergency medicine.” Moreover, further research is needed on the effects of improving the emergency medical care system by organically integrating “ER-type emergency medicine” with the “emergency medical center-emergency medical care model” and “department-specific emergency medical care model,” which are complementary to each other. Consequently, a new and better emergency medical care system can be created for regions and countries where ER-emergency medicine is not widespread.

A histogram of the distribution by beds of the facilities that distributed the survey and those that actually responded shows a trend toward more responses from facilities with more beds than the actual distribution (not shown). Of the responding facilities, Hospitals with 199 beds or less accounted for 62%, making up the largest group (Fig. 2). It is generalizable in similar emergency medical care systems but not applicable in other systems, national or regional. However, some of the free-text content could exist in similar opinions in any EMS agency, regardless of the system.

### Limitations

One limitation of this survey is that the response rate was only 34% (1289 facilities). Selection bias based on the responding facilities (higher response rates were observed from hospitals with more beds) could be involved. Contrarily, it was possible to include responses from emergency medical institutions with several beds. Nevertheless, the results must be interpreted with caution because of the many limitations of the survey method employed.

## Conclusion

This is the first large-scale survey on the status and issues of emergency medical care systems at secondary emergency medical institutions in Japan. The results of this survey are expected to be considered during the formulation of policies of the Japanese Society of Emergency Medicine and other related academic societies, governments, local medical planning, and medical control. Based on the results of this survey, the JAAM and core regional facilities, national and local governments should consider and implement specific measures for regions and hospitals with few emergency medicine specialists and physicians (e.g., development of ER-based emergency medicine and provision of education). This will lead to the improvement of the emergency medical care system that supports the region and the establishment of a new emergency medical care system that society demands.

## Methods

### Study design

This observational study utilized questionnaires.

### Setting

All 4063 emergency medical facilities in Japan (certified by prefectural governors based on the Ministerial Ordinance Establishing Emergency Hospitals, and so on [Ministry of Health and Welfare Ordinance No. 8, 1964]) were included in the survey (Fig. 1). Tertiary hospitals were excluded from the study. Only the responding secondary emergency hospitals were included in the final analysis.

### Ethical considerations

This study was performed in accordance with the ethical standards as laid down in the 1964 Declaration of Helsinki and its later amendments or comparable ethical standards, and it was approved by the Ethics Review Committee of Hiroshima Prefectural Hospital (no. 202101–5; approval date: January 27, 2021). This study was also approved by the Board of Directors of the JAAM and was conducted mainly by the Study Committee of the Emergency Department of the JAAM. Participants’ responses constituted their consent for participation.

### Survey items and methodology

A survey form (Table 5) was distributed by mail to 4063 emergency medical facilities (including 287 tertiary emergency centers) in Japan. Most hospitals were secondary emergency hospitals (93%). Information from January 1, 2019, to March 31, 2021, was elicited, and responses were collected both on paper and online. To avoid duplicate responses and to confirm the content of the information, the names of respondents were collected. Medical institutions with emergency centers were excluded from the analysis, given the study purpose. Blank cells and values that were misdescribed were considered missing values.

**Table 5:**
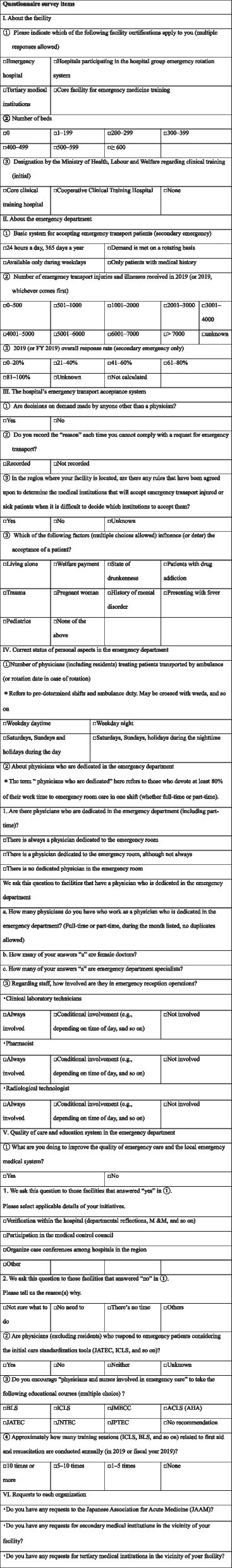
Questionnaire survey items

*BLS* basic life support, *ICLS* immediate cardiac life support, *JMECC* Japanese Medical Emergency Care Course, *ACLS (AHA)* Advanced Cardiovascular Life Support (American Heart Association), *JATEC* Japan Advanced Trauma Evaluation and Care, *JNTEC* Japan Nursing for Trauma Evaluation and Care, *JPTEC* Japan Prehospital Trauma Evaluation and Care

For “81% or more annual response rate,” cross-tabulation was conducted based on whether the institution was “designated as a clinical training hospital;” “has full-time physicians;” “has pharmacists, clinical technologists, and radiologists available at all times;” “is making efforts to improve the quality of emergency care and the local emergency medical system;” “has 200 or more beds;” and “is a core facility of an emergency department training program.” Cross-tabulation was conducted on whether the facility was a “core facility of the emergency medicine specialty training program.”

The following items were also cross-tabulated according to whether the hospital was designated as a clinical training hospital: “keeps records of reasons for failure to respond to requests for admission;” “always has a full-time physician available;” “pharmacists, clinical laboratory technicians, and radiology technicians are always available;” “is working to improve the quality of emergency care and the local emergency medical system;” and “is taking steps to standardize initial treatment by doctors treating emergency patients.” The results were cross-tabulated according to whether the hospital was designated a clinical training hospital. The “number of full-time physicians” and “number of female physicians among full-time physicians” were compared based on whether the hospital was designated as a clinical training hospital or not (Mann–Whitney *U* test). Logistic regression analysis was also performed, with the objective variable being whether the facility had an “annual response rate of 81% or more.”

The free-text responses were reviewed and categorized based on keywords. To understand the characteristics of the facilities that gave specific answers in the free-response columns, the following items were used: “designated hospital for clinical training,” “core facility of emergency department specialty training program,” “rotating participating hospital,” “more than 200 beds,” “annual demand rate of 81% or more” (each item listed by demand rate), and “making some efforts to improve quality of emergency care and local emergency medical system.” All statistical analyses were performed using SPSS 28.0 (IBM SPSS Statistics; IBM, Chuo-ku, Tokyo, JAPAN). Significance was set at *P* < 0.05.

## Data Availability

Owing to the nature of this study, the participants involved have not agreed to these data being shared publicly; consequently, supporting data are not available. Additionally, these data belong to the JAAM committee.

## Abbreviations

BLS: Basic life support
EMS: Emergency medical services
ER: Emergency medicine
ICLS: Immediate Cardiac Life Support
JATEC: Japan Advanced Trauma Evaluation and Care
